# The effects of smoking on the dynamic vocal range of male smokers

**DOI:** 10.1590/2317-1782/e20240325en

**Published:** 2026-02-27

**Authors:** Lídia Cristina da Silva Teles, Mariana Ferreira Gonçalves, Joice Aparecida Costa Bernardo, Kemellyn Nayara Veiga

**Affiliations:** 1 Departamento de Fonoaudiologia, Faculdade de Odontologia de Bauru – FOB, Universidade de São Paulo – USP - Bauru (SP), Brasil.; 2 Programa de Pós-graduação Interunidades em Bioengenharia, Escola de Engenharia de São Carlos – EESC, Instituto de Química de São Carlos – IQSC, Faculdade de Medicina de Ribeirão Preto – FMRP, Universidade de São Paulo – USP - São Carlos (SP), Brasil.; 3 Hospital Estadual de Ribeirão Preto - Ribeirão Preto (SP), Brasil.; 4 Centro Integrado de Reabilitação, Prefeitura de Tatuí - Tatuí (SP), Brasil.

**Keywords:** Voice, Acoustics, Tobacco Smoking, Public Health, Speech, Language and Hearing Sciences

## Abstract

**Purpose:**

To investigate the effects of tobacco use, smoking duration, and number of cigarettes/day on the vocal dynamic field of male smokers.

**Methods:**

A cross-sectional, comparative observational study was conducted with 62 men aged 18 to 59 years: 31 smokers (SG) and 31 non-smokers (NSG). Vocal dynamic field measurements were obtained using phonetography, which assesses frequency (minimum, maximum, and vocal range) and intensity (minimum, maximum, and maximum dynamic range). Comparisons between groups were made using the Student’s t-test, and correlations with smoking duration and number of cigarettes/day were analyzed using Pearson’s correlation test (5% significance level).

**Results:**

Compared to the NSG, the SG showed significantly lower values (p < 0.05) for maximum frequency, vocal range, and maximum dynamic range, and higher values for minimum intensity. Increased smoking duration was associated with reductions in minimum and maximum frequencies, vocal range, and maximum intensity. Additionally, a higher number of cigarettes/day was correlated with lower minimum frequency.

**Conclusion:**

The dynamic field of male smokers, compared to non-smokers, showed a reduction in the values of maximum frequency, vocal range and maximum dynamic range and an increase in minimum intensity. The longer the time of tobacco use, the lower the minimum and maximum frequencies, vocal range and maximum intensity. The greater the number of cigarettes/day, the lower the value of minimum frequency. Phonetography is an effective resource in voice assessments.

## INTRODUCTION

The smoking habit spread across Europe and the Americas after the First World War. In the United States, cigarettes were widely promoted through advertising as a symbol of elegance and sophistication, and in certain contexts, as a form of social acceptance. However, smoking has evolved into a silent and growing epidemic, constituting a serious public health problem because it contradicts the principles of health promotion. As such, this practice has been widely discouraged and restricted^(1)^.

According to recent estimates by the World Health Organization (WHO)^(2)^ and the Pan American Health Organization (PAHO)^(3)^, approximately 1.25 billion people worldwide consumed tobacco in 2024, 1 billion of whom were men.Tobacco is a significant risk factor for six of the eight leading causes of death globally, including acute myocardial infarction, stroke, respiratory infections, and cancers of the lung, larynx, and esophagus, contributing to one in ten deaths among adults^(3-6)^. WHO^(2)^ and PAHO^(3)^ also highlight that more than seven million deaths annually are attributed to direct tobacco use, with 80% of these occurring in low- and middle-income countries, which are heavily impacted by the influence of the tobacco industry.

The larynx is particularly vulnerable to the harmful effects of smoking and may present with chronic inflammation of the mucosa, leukoplakia, erythema, hyperkeratosis, metaplasia, and hyperplasia^(7-8)^. Studies indicate that the vocal quality of smokers is often described as hoarse-crackling, hoarse-breathy, and strained^(9)^. Furthermore, the literature highlights the impact of tobacco on acoustic measures of the voice, including the reduction of fundamental frequency and vocal intensity ^(10-11)^.

Scientific evidence suggests that vocal quality and respiratory physiology are impaired by the duration of smoking, as reported in the literature ^(10,12-14)^. In addition, the daily amount of cigarettes consumed also has a significant impact on these functions, as described by Wei et al.^(14)^ and Silva et al.^(15)^. These findings reinforce the dose-dependent relationship between smoking and vocal and respiratory impairment.

The dynamic field of voice, also called “voice range profile”, can be obtained through phonetography, an examination that evaluates vocal intensity measurements at maximum and minimum limits throughout the entire vocal range^(16-20)^. The dynamic field of voice is recorded in the phonetogram, a phonetography graph, which constitutes an important visual feedback resource for the patient^(16-20)^. Phonetography has been used in studies with different populations, such as elderly women^(16)^, child and adolescent singers^(17)^, in the standardization of epidemiological studies^(18)^, in the development of normative data for young women^(19)^, and in the analysis of the passing note of choir singers through the identification of frequency and intensity^(20)^. These studies demonstrate that phonetography allows a comprehensive assessment of vocal ability, as well as being a useful tool for monitoring therapeutic progress.

The hypothesis of the present study postulates that smoking reduces the dynamic field of voice in one or more measures of frequency and/or vocal intensity of male smokers, compared to non-smoking men. Furthermore, prolonged tobacco use and a higher daily number of cigarettes are expected to have a negative impact on the dynamic field of voice.

To date, the literature lacks studies that specifically investigate the effects of tobacco on the dynamic field of voice. Given this gap, the present study aims to evaluate the impacts of smoking—including the influence of the duration of tobacco use and the daily quantity of cigarettes consumed—on the dynamic field of voice of male smokers.

## METHODS

### Type of study

This is an observational, cross-sectional, and comparative study.

### Ethical considerations

This study was approved by the Research Ethics Committee under number 1.526.594.

### Sample

The participants in this study were recruited through social media platforms.

The inclusion criterion established for the smokers group (SG) was: being 18 years of age or older and having been a conventional cigarette smoker for more than five years.

The inclusion criteria for participants in the non-smoking group (NSG) were: not being and never having been a smoker, in addition to having an age corresponding to that of a participant in SG, following a 1:1 proportionality.

The exclusion criteria for both groups were: being 60 years of age or older; having respiratory diseases on the day of the examination; having undergone laryngeal surgery; having hearing complaints; being or having been a voice professional; and having or having had exposure to chemical agents in the workplace. These criteria were established to minimize confounding variables and ensure that the study results specifically reflected the effects of smoking on the voice, without the interference of other conditions that could impact vocal quality and health^(21)^.

For inclusion in the sample, participating smokers answered a questionnaire about the exclusion criteria.

62 adult Brazilian men, divided into two groups, participated:

- Smoking group: 31 men, aged between 20 and 58 years, mean age 35 years and 10 months (SD = 21 years and 11 months).
- Non-smoking group: 31 men, aged between 18 and 59 years, mean age 36 years and 8 months (SD = 23 years and 4 months).

The equivalence of ages between the SG and NSG groups was verified by the paired t-test, with a significance level of 5%, and the result did not indicate a difference between the ages (p=0.7823).

### Procedures

#### The place

The phonetographies of all participants were performed in the voice studio of the Speech-Language Pathology and Audiology Clinic of the Bauru School of Dentistry, University of São Paulo, in a room with acoustic treatment^(22)^, whose infrastructure allows the reduction of ambient noise to 43dB SPL, guaranteeing adequate conditions for the recordings.

#### Phonetography

Phonetography allows for the evaluation of the dynamic field of voice, encompassing the physiological limits of frequency and intensity. The examination results in a two-dimensional graph, called a phonetogram, which allows for the analysis of the individual's vocal frequency and intensity limits^(16-19)^.

The 62 selected participants underwent a phonetography examination in the voice laboratory using the Voice Range Profile (VRP) program from Multi Speech, by Kay Pentax.

The examination was conducted with the participant seated, with their spine erect, using an AKG C44 unidirectional headset microphone positioned 3 cm from the corner of the mouth.

**Figure 1:**
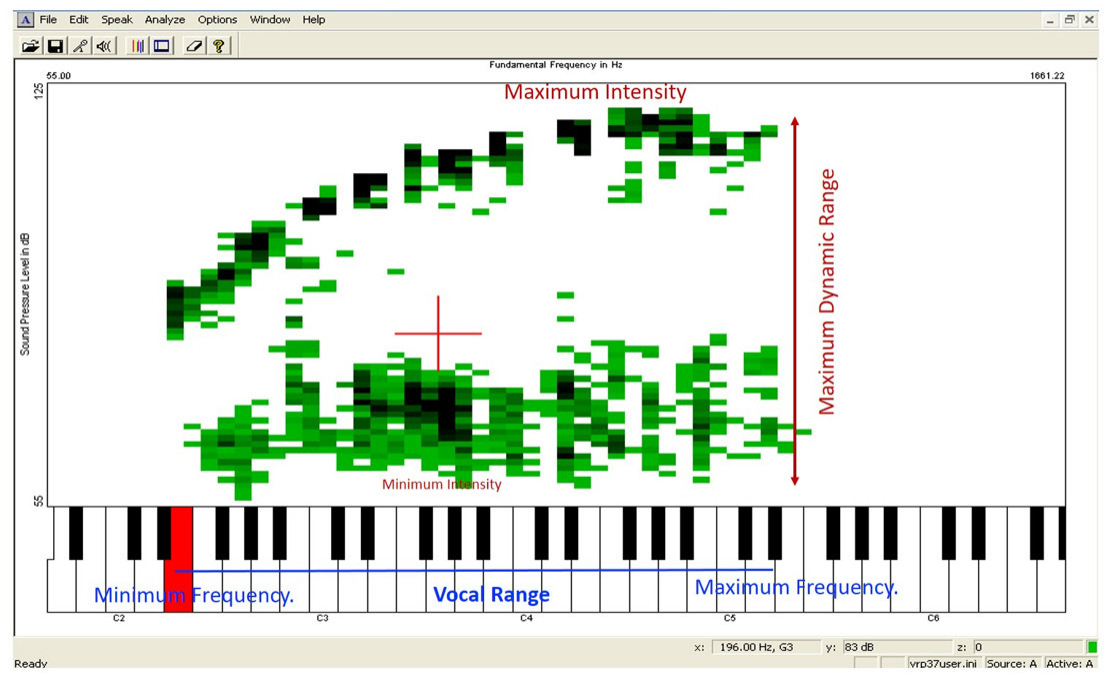
Screenshot of the phonogram extracted from the Vocal Range Profile program by Kay Pentax. Frequencies are represented on the horizontal axis and intensities on the vertical axis. Frequency measurements are indicated in blue and intensity measurements in red (inserted by the authors)

To perform the phonetography, participants were instructed to produce the vowel /a/, following the note given by the evaluator. The emission should be sustained for at least five seconds, initially at the lowest possible intensity, and then at the highest possible intensity^(16)^. The test began at the note C3 (131 Hz), a vocal comfort note for men. From there, the notes C, E, G, and A were emitted in an ascending scale until reaching the maximum frequency, the maximum limit of the participant's vocal range. Subsequently, repeating the note C3, the notes C, A, G, and E were presented in descending order until the lowest possible frequency for the participant was reached, the minimum limit of their vocal range. All vocalizations, at all frequencies and minimum and maximum intensities, were recorded in the program interface, which generated a graph called a phonetogram (Figure 1), in which frequencies are represented on the abscissa (horizontal axis) and intensities on the ordinate (vertical axis). The total duration of the examination was approximately 30 minutes.

The phonetograms were analyzed to obtain the following parameters according to Teles-Magalhães et al.^(16)^ (Figure 1):

#### Frequency measurements: obtained on the horizontal axis of the phonetogram

- Minimum frequency: expressed in Hertz (Hz) and semitones (st), it corresponds to the lowest note produced by the participant.
- Maximum frequency: expressed in Hertz (Hz) and semitones (st), it corresponds to the highest note achieved by the participant.
- Vocal range: expressed in semitones (st), it represents the number of semitones produced between the minimum and maximum frequencies.

#### Intensity measurements: obtained on the vertical axis of the phonetogram.

- Minimum intensity: expressed in decibels (dB), corresponds to the lowest intensity recorded among the minimum intensities produced throughout the vocal range (lower curve of the phonetogram).
- Maximum intensity: expressed in decibels (dB), corresponds to the highest intensity recorded among the maximum intensities produced throughout the vocal range (upper curve of the phonetogram).
- Maximum dynamic range (MDR): expressed in decibels (dB), represents the greatest difference between the minimum and maximum intensities at the same frequency.

The upper and lower curves of the phonetogram determine the dynamic range or voice range profile of the participants. In Figure 1, it can be seen that the green registers correspond to the recorded vocal emissions of the participants. When the participant repeats the same utterance, the points overlap, resulting in a dark green hue. The most important aspect is the recording of intensities at the maximum (upper curve) and minimum (lower curve) limits across the frequencies throughout the vocal range. Therefore, during the phonetography examination, the individual doesn't need to produce emissions in the central region of the dynamic range, since all emissions below the upper curve and above the lower curve are considered to belong to their voice dynamic range.

### Data analysis

The data obtained from the phonetograms of participants in SG and NSG groups were analyzed using percentages, means, and standard deviations.

The normality of the parameters extracted from the phonetogram was determined using the Shapiro-Wilk test.

The comparison between the SG and NSG groups, for each phonetography parameter, was performed using the Student's t-test for independent samples, adopting a significance level of 5%.

Furthermore, correlations were performed between the duration of tobacco use and the number of cigarettes consumed per day, with the parameters obtained by phonetography, using Pearson's correlation test, also with a significance level of 5%.

## RESULTS

### Sample characterization

The characterization of the participants in the SG and NSG groups is shown in Table 1. It should be noted that the smoking duration of the SG participants ranged from 5 to 46 years, and the number of cigarettes smoked per day ranged from 5 to 30 cigarettes.

**Table 1:** Descriptive analysis of participants in the Smoking Group (SG) and the Non-Smoking Group (NSG)

**Smoking Group**				**Non-Smoking Group**	
**Nº**	**Age (years)**	**Tobacco use (years)**	**NºCigarretes/day**	**N**	**Age (years)**
1	20	5	5	1	18
2	21	5	20	2	23
3	21	6	7	3	21
4	23	6	20	4	21
5	24	7	15	5	26
6	21	7	20	6	21
7	23	8	10	7	22
8	25	8	10	8	23
9	26	8	10	9	25
10	27	8	20	10	26
11	28	10	10	11	28
12	26	10	20	12	24
13	27	11	10	13	26
14	33	14	5	14	34
15	29	15	20	15	30
16	32	15	20	16	32
17	31	17	20	17	32
18	31	18	10	18	30
19	47	18	20	19	45
20	48	20	5	20	49
21	40	22	5	21	39
22	37	22	15	22	37
23	50	24	5	23	50
24	41	24	10	24	42
25	57	24	15	25	58
26	54	34	15	26	54
27	49	35	15	27	49
28	50	35	30	28	52
29	54	38	12	29	54
30	58	43	20	30	57
31	58	46	20	31	59
**Mean (SD)**	35 years and 10 months (21 years and 11 months)	18 years and 6 months (12 years and 10 months)	14 (6)		36 years and 8 months (23 years and 4 months)

**Caption:** nº = number of participants; SD = standard deviation

The results showed a strong positive correlation between the age of the participants in the SG and the duration of tobacco use (coefficient = 0.929; p = 0.0001), indicating that older age is directly associated with a longer duration of smoking.

On the other hand, no correlation was identified between the age of the participants and the number of cigarettes consumed per day (coefficient = 0.156; p = 0.3934).

Similarly, the duration of tobacco use did not show a correlation with the number of cigarettes smoked per day (coefficient = 0.218; p = 0.3196).

### Phonetography

The parameters analyzed by the Shapiro-Wilk normality test indicated that the data extracted from the phonetogram follow a normal distribution, as shown in Table 2.

**Table 2:** Distribution of Phonetrography variables, p-value and Shapiro-Wilk Normality Test conclusion

	Variables of Phonography	p	Test Conclusion
**Frequencies**	Minimum Frequency(Hz)	0.132	Normal
	Maximum Frequency(Hz)	0.338	Normal
	Vocal Extension	0.585	Normal
			
**Intensities**	Minimum Intensity (dB)	0.095	Normal
	Maximum Intensity (dB)	0.085	Normal
	Maximum Dynamic Range (dB)	0.250	Normal

**Caption:** Hz = Hertz; dB = Decibel

The participants in SG, when compared to those in the NSG, presented lower values in frequency measurements (maximum and vocal range). Regarding intensity measurements, a lower value was observed in the maximum dynamic range and a higher value in the minimum intensity (Table 3).

**Table 3:** Mean values and standard deviations of intensity and frequency measurements obtained by phonetography of participants in the smoking (SG) and non-smoking (NSG) groups, and a comparison between the groups

PHONETOGRAPHY MEASURES		SG Mean (SD)	NSG Mean (SD)	p Value
	Minimum (Hz)	84.3 (14.3)	86.4 (16.7)	0.696
**Frequencies**	**Maximum (Hz)**	518.2 (166.3)	714.1 (262.8)	**0.004** ^*^
	**Vocal Extension (st)**	31.5 (6.3)	36.9 (9.0)	0.005*
				
	**Minimum (dB)**	66.4 (7.3)	62.3 (5.7)	0.016*
**Intensities**	**Maximum (dB)**	114.9 (6.4)	117.4 (6.6)	0.127
	**Maximum Dynamic Range (dB)**	45.0 (9.4)	54.0 (9.2)	0.003*

* Significant values (p<0.05) - Student's t-test for two independent samples

**Caption:** SD = Standard deviation; Hz = Hertz; St = Semitones; dB = Decibel

**Figure 2:**
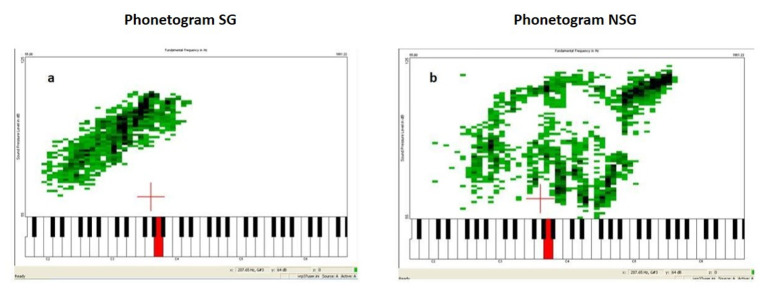
Screenshots of phonetograms from two study participants: a) Phonetogram of a participant from the Smoking Group (SG), 58 years old, smoker for 43 years, with a daily consumption of 20 cigarettes. b) Phonetogram of a participant from the Non-Smoking Group (NSG), 59 years old

Figure 2 illustrates the comparison between the phonetogram of a participant from the SG and that of a NSG participant, both of similar ages.

In the correlation analysis between the duration of tobacco use and the number of cigarettes per day with the phonetography parameters of the participants in SG, a positive correlation was observed between the duration of smoking and all frequency measures (minimum, maximum, and vocal range), as well as with the minimum intensity. Regarding the number of cigarettes consumed per day, a positive correlation was identified only with the minimum frequency (Table 4).

**Table 4:** Correlation of tobacco smoking duration and number of cigarettes per day with the parameters of frequency and intensity of phonetography of participants in the Smoking Group (SG).

	Frequencies			Intensities		
	Minimum	Maximum	Vocal Extension	Minimum	Maximum	Maximum Dynamic Range
**Time Tobacco**	**-0.32 (0.024)** ^*^	-0.31 (0.006)*	-0.39 (0.043)*	0.03 (0.860)	-0.40 (0.033)*	-0.34 (0.908)
**Number of Cigarettes/day**	-0.45 (0.016)*	0.07 (0.933)	0.21 (0.322)	0.13 (0.477)	0.01 (0.805)	-0.01 (0.943)

* Significant values (p<0.05) - Pearson correlation test

## DISCUSSION

This study sought to investigate the effects of tobacco use, smoking duration, and number of cigarettes smoked per day on the dynamic field of voice of male smokers using phonetography. The results confirmed the initially proposed hypothesis. However, no previous studies were found that used this tool specifically with smokers.

In the phonetographic analysis, the reduction in maximum frequency values and vocal range observed in participants in the SG, compared to those in the NSG, can be attributed to the impact of smoking on vocal fold tissue. Ayoub et al.^(10)^ and Pinto et al.^(22)^ reported a decrease in the fundamental frequency in the voices of smokers, resulting from increased vocal fold mass. This change limits the range of motion of the vocal folds, resulting in a reduced ability to produce sounds at higher frequencies.

Regarding the intensity measurements obtained through phonetography, a significant increase in minimum intensity and a reduction in maximum dynamic range were observed in SG compared to the NSG. This finding suggests that smokers have difficulty maintaining vocal fold vibration at the extremes of intensity, which may be attributed to the decreased aerodynamic forces of phonation, associated with lung changes and respiratory impairment caused by smoking^(12)^. According to Boskabady et al.^(12)^, cigarettes impact the vocal control of smokers.

The wide variation in smoking duration among the SG participants (5 to 46 years) was relevant for the analysis of the correlation between smoking duration and phonetography parameters. The negative correlation observed between smoking duration and all frequency-related parameters indicated that the longer the smoking duration, the greater the shift of minimum and maximum frequencies to a lower register and the smaller the vocal range. These results corroborate findings in the literature^(10,14)^ that reported a reduction in fundamental frequency and cepstral peak due to thickening and inflammation of the vocal fold mucosa.

In the correlation between smoking time and phonetography intensity measurements, a negative correlation was observed between smoking time and maximum intensity. This result corroborates studies^(12,15)^ conducted with spirometry in smokers, which indicated that the habit of smoking induces chemical dependence and, with prolonged use, compromises respiratory capacity, increasing expiratory effort. The authors^(12,15)^ justified that the influence of cigarette toxins, accumulated over time, reduces the strength of expiratory airflow, necessary to maintain intensity at its maximum limit.

In the analysis of the correlation between the number of cigarettes consumed per day (5 to 30 cigarettes/day) and the phonetography measurements, a negative correlation was observed between the number of daily cigarettes and the minimum frequency. The greater the number of cigarettes consumed per day, the lower the minimum vocal frequency. It is known that a reduction in vocal frequency is related to an increase in the mass of the vocal folds. Therefore, it can be inferred that higher daily cigarette consumption can cause edema and thickening of the vocal fold mucosa. A study that evaluated the increase in smoking volume showed worse results in the cepstral peak analysis in vocal acoustic evaluation^(14)^.

The average of 14 cigarettes per day among the SG participants was similar to the averages reported in studies with Iranian smokers^(12)^ and American smokers^(23)^ — 14 and 17 cigarettes per day, respectively — indicating a high rate of cigarette consumption in these populations. In the present study, it is noteworthy that neither age nor duration of smoking influenced the number of cigarettes consumed. It is noteworthy that, although cigarettes are addictive^(15)^, the duration of smoking had no impact on the number of cigarettes smoked per day.

One of the limitations of the present study was the lack of verification of the history of exposure to secondhand smoke, since the possibility of the participants in the control group frequenting environments with smokers—a factor that could influence vocal quality—was not considered. It is suggested that future studies include and analyze this variable.

As a recommendation for future studies, it is suggested to verify the voice dynamic range in other types of cigarettes, such as hand-rolled and electronic cigarettes. It is also suggested to specify the race of the participants, since few studies consider vocal variation according to race.

Disseminating information about the impact of smoking on the voice dynamic range could play a crucial role in expanding the knowledge of speech-language pathologists and audiologists, and professionals working in vocal health. In addition to the well-established risk of laryngeal cancer and other pathologies caused by conventional cigarette smoking, as well as the harmful effects on the voice, the evidence of a reduction in the voice dynamic range in voice professionals—especially singers—is highly relevant, since these professionals frequently use their voices at the maximum limits of this range.

Phonetography proved to be a useful tool as a complementary instrument in voice assessments ^(16-20)^, especially in the population studied. Its results confirmed the vocal changes associated with smoking, corroborating the findings in the literature obtained through other assessment methods.

## CONCLUSION

The vocal dynamic range of **male smokers** showed: 1) significant changes compared to **non-smoking men** - lower values for maximum frequency, vocal range, and maximum dynamic range, and higher values for minimum intensity; 2) the longer the smoking time, the lower the values for minimum and maximum frequencies, vocal range, and maximum intensity; and 3) the greater the number of cigarettes consumed per day, the lower the value for minimum frequency.

Phonetography has proven to be a useful tool as a complementary instrument in voice assessments.
