# Supplementary material for: The effects of smoking on the dynamic vocal range of male smokers
Source: Codas. 2026 Feb 27;38(1):e20240325. doi: 10.1590/2317-1782/e20240325en (PMC12978333; doi:10.1590/2317-1782/e20240325en)
Supplement: Translation

# Translation: Efeitos do cigarro no campo dinâmico da voz de homens fumantes

**DOI:** 10.1590/2317-1782/e20240325pt

**Authors:** Lídia Cristina da Silva Teles, Mariana Ferreira Gonçalves, Joice Aparecida Costa Bernardo, Kemellyn Nayara Veiga

**Affiliations:** 1 Departamento de Fonoaudiologia, Faculdade de Odontologia de Bauru – FOB, Universidade de São Paulo – USP - Bauru (SP), Brasil.; 2 Programa de Pós-graduação Interunidades em Bioengenharia, Escola de Engenharia de São Carlos – EESC, Instituto de Química de São Carlos – IQSC, Faculdade de Medicina de Ribeirão Preto – FMRP, Universidade de São Paulo – USP - São Carlos (SP), Brasil.; 3 Hospital Estadual de Ribeirão Preto - Ribeirão Preto (SP), Brasil.; 4 Centro Integrado de Reabilitação, Prefeitura de Tatuí - Tatuí (SP), Brasil.

**Keywords:** Voz, Acústica, Fumar Tabaco, Saúde Pública, Fonoaudiologia

## Abstract

**Objetivo:**

Investigar os efeitos do tabaco, a influência do tempo de fumo e da quantidade de cigarros/dia no campo dinâmico da voz de homens fumantes.

**Método:**

Estudo observacional transversal e comparativo. Participaram 62 homens entre 18 e 59 anos: 31 no grupo fumantes (GF) e 31 no grupo de não fumantes (GNF). O campo dinâmico da voz dos participantes foi obtido pela fonetografia que registra medidas de frequência (mínima, máxima e extensão vocal) e de intensidade (mínima, máxima e extensão dinâmica máxima). As medidas da fonetografia dos participantes dos GF foram comparadas com as do GNF pelo Teste t Student e correlacionadas com o tempo de fumo e com número de cigarros por dia pelo Teste de correlação de Pearson, nível de significância 5%.

**Resultados:**

O GF apresentou valores menores (p<0,05) em relação ao GNF na frequência máxima, extensão vocal e extensão dinâmica máxima e valor maior na intensidade mínima. O tempo maior de fumo reduziu as frequências mínima e máxima, a extensão vocal e a intensidade máxima. A quantidade maior de cigarros/dia diminuiu a frequência mínima.

**Conclusão:**

O campo dinâmico dos homens fumantes, comparados a homens não fumantes, apresentou redução dos valores de frequência máxima, extensão vocal e extensão dinâmica máxima e aumento da intensidade mínima. Quanto maior o tempo de uso do tabaco, menores as frequências mínima e máxima, a extensão vocal e a intensidade máxima. Quanto maior a quantidade de cigarros/dia, menor o valor da frequência mínima. A fonetografia é um recurso eficaz nas avaliações da voz.

## INTRODUÇÃO

O hábito de fumar disseminou-se na Europa e nas Américas a partir da Primeira Guerra Mundial. Nos Estados Unidos, o cigarro foi amplamente promovido pela publicidade como símbolo de elegância, sofisticação e, em determinados contextos, como uma forma de aceitação social. Contudo, o tabagismo evoluiu para uma epidemia silenciosa e crescente, configurando-se como um grave problema de saúde pública por contrariar os princípios da promoção da saúde. Nesse sentido, essa prática tem sido amplamente desaconselhada e restrita^(1)^.

Segundo estimativas recentes da Organização Mundial da Saúde (OMS)^(2)^ e da Organização Pan-Americana da Saúde (OPAS)^(3)^, em 2024, aproximadamente 1,25 bilhão de pessoas no mundo consomem tabaco, sendo 1 bilhão delas homens. O tabaco é um fator de risco significativo para seis das oito principais causas de morte globalmente, incluindo infarto agudo do miocárdio, acidente vascular encefálico, infecções respiratórias e cânceres de pulmão, laringe e esôfago, contribuindo para uma em cada dez mortes entre adultos^(3-6)^. A OMS^(2)^ e a OPAS^(3)^ destacam ainda que mais de sete milhões de mortes anuais são atribuídas ao uso direto do tabaco, sendo que 80% dessas ocorrem em países de baixa e média renda, fortemente impactados pela influência da indústria tabagista.

A laringe é particularmente vulnerável aos efeitos nocivos do tabagismo, podendo apresentar inflamação crônica da mucosa, leucoplasia, eritema, hiperceratose, metaplasia e hiperplasia^(7-8)^. Estudos indicam que a qualidade vocal de fumantes é frequentemente descrita como rouca-crepitante, rouca-soprosa e tensa^(9)^. Além disso, a literatura evidencia o impacto do tabaco em medidas acústicas da voz, incluindo a redução da frequência fundamental e da intensidade vocal^(10-11)^.

Evidências científicas demonstram que a qualidade vocal e a fisiologia respiratória são prejudicadas pela duração do hábito de fumar, conforme relatado na literatura^(10,12-14)^. Ademais, a quantidade diária de cigarros consumidos também exerce impacto significativo sobre essas funções, como descrito por Wei et al.^(14)^ e Silva et al.^(15)^. Esses achados reforçam a relação dose-dependente entre o tabagismo e o comprometimento vocal e respiratório.

O campo dinâmico da voz, também denominado “perfil da extensão vocal”, pode ser obtido por meio da fonetografia, exame que avalia as medidas de intensidade vocal nos limites máximos e mínimos ao longo de toda a extensão vocal^(16-20)^. O campo dinâmico vocal é registrado no fonetograma, gráfico da fonetografia, que constitui um importante recurso de feedback visual para o paciente^(16-20)^. A fonetografia tem sido utilizada em estudos com diferentes populações, como em mulheres idosas^(16)^, crianças e adolescentes cantores^(17)^, na padronização para estudos epidemiológicos^(18)^, no desenvolvimento de dados normativos para mulheres jovens^(19)^ e na análise da nota de passagem de coristas por meio da identificação da frequência e da intensidade^(20)^. Esses estudos demonstram que a fonetografia permite uma avaliação abrangente da capacidade vocal, além de ser uma ferramenta útil para o monitoramento do progresso terapêutico.

A hipótese do presente estudo postula que o tabagismo reduz o campo dinâmico da voz em uma ou mais medidas de frequência e/ou intensidade vocal de homens fumantes, em comparação a homens não fumantes. Ademais, espera-se que o tempo prolongado de uso do tabaco e a maior quantidade diária de cigarros tenham impacto negativo sobre o campo dinâmico vocal.

Até o momento, a literatura carece de estudos que investiguem especificamente os efeitos do tabaco sobre o campo dinâmico da voz. Diante dessa lacuna, o presente estudo tem como objetivo avaliar os impactos do tabagismo — incluindo a influência da duração do uso do tabaco e da quantidade diária de cigarros consumidos — no campo dinâmico da voz de homens fumantes.

## MÉTODO

### Tipo do estudo

Este é um estudo observacional, transversal e comparativo.

### Considerações éticas

Este estudo foi aprovado pelo Comitê de Ética em Pesquisa sob o parecer número 1.526.594.

### Amostra

Os participantes deste estudo foram recrutados por meio de plataformas de redes sociais.

O critério de inclusão estabelecido para o grupo de fumantes (GF) foi: ter 18 anos ou mais e ser tabagista de cigarro convencional há mais de cinco anos.

Os critérios de inclusão para os participantes do grupo de não fumantes (GNF) foram: não ser e nunca ter sido fumante, além de ter idade correspondente à de um participante do grupo de fumantes (GF), seguindo a proporcionalidade 1:1.

Os critérios de exclusão para ambos os grupos foram: ter idade igual ou superior a 60 anos; apresentar doenças respiratórias no dia do exame; ter sido submetido a cirurgias laríngeas; ter queixas auditivas; ser ou ter sido profissional da voz; e ter ou ter tido exposição a agentes químicos no ambiente de trabalho. Esses critérios foram estabelecidos para minimizar variáveis de confusão e garantir que os resultados do estudo refletissem especificamente os efeitos do tabagismo na voz, sem a interferência de outras condições que pudessem impactar a qualidade e saúde vocal^(21)^.

Para inclusão na amostra, os participantes fumantes responderam a um questionário referente aos critérios de exclusão.

Participaram 62 homens adultos brasileiros, distribuídos em dois grupos:

- Grupo de fumantes (GF): 31 homens, com idades entre 20 e 58 anos, média de 35 anos e 10 meses (DP = 21 anos e 11 meses).
- Grupo de não fumantes (GNF): 31 homens, com idades entre 18 e 59 anos, média de 36 anos e 8 meses (DP = 23 anos e 4 meses).

A equivalência das idades entre os grupos GF e GNF foi verificada pelo teste estatístico t pareado, com nível de significância de 5% e o resultado não indicou diferença entre as idades (p=0,7823).

### Procedimentos

#### Local do estudo

As fonetografias de todos os participantes foram realizadas no estúdio de voz da Clínica de Fonoaudiologia da Faculdade de Odontologia de Bauru da Universidade de São Paulo, uma sala com tratamento acústico^(22)^, cuja infraestrutura permite a redução do ruído ambiente para 43dB NPS, garantindo condições adequadas para as gravações.

#### Fonetografia

A fonetografia possibilita a avaliação do campo dinâmico da voz, abrangendo os limites fisiológicos de frequência e intensidade. O exame traz como resultado um gráfico bidimensional, denominado fonetograma, que permite a análise dos limites vocais da frequência e da intensidade do indivíduo^(16-19)^.

Os 62 participantes selecionados realizaram no laboratório de voz, o exame de fonetografia utilizando o programa Voice Range Profile (VRP) da Multi Speech, da Kay Pentax.

O exame foi conduzido com o participante sentado, com a coluna ereta, utilizando um microfone de cabeça unidirecional, modelo C44 da AKG, posicionado a uma distância de 3 cm da comissura labial.

Para a realização da fonetografia, os participantes foram instruídos a produzir a vogal /a/, seguindo a nota emitida pelo avaliador. A emissão deveria ser feita de forma sustentada, por no mínimo cinco segundos, inicialmente na menor intensidade possível, e em seguida na maior intensidade possível^(16)^. O teste teve início na nota C3 (131 Hz), nota de conforto vocal para homens. A partir dela foram emitidas as notas dó, mi, sol e lá (C, E, G e A) em escala ascendente, até alcançar a frequência máxima, limite máximo da extensão vocal do participante. Posteriormente, repetindo a nota C3, foram apresentadas em escala descendente as notas dó, lá, sol, mi (C, A, G e E) até a menor frequência possível para o participante, limite mínimo da extensão vocal. Todas as emissões, em todas as frequências nas intensidades mínimas e máximas, foram registradas na interface do programa, que gerou um gráfico denominado fonetograma (Figura 1), no qual as frequências estão representadas na abscissa (eixo horizontal) e as intensidades na ordenada (eixo vertical). A duração total do exame foi de aproximadamente 30 minutos.

**Figura 1:** Imagem da tela do Fonetograma extraída do Programa Vocal Range Profile, da Kay Pentax. As frequências estão representadas no eixo horizontal e as intensidades no eixo vertical. As medidas de frequência estão indicadas em azul e as de intensidade em vermelho (inseridas pelos autores)

Os fonetogramas foram analisados para obtenção dos seguintes parâmetros de acordo com Teles-Magalhães et al.^(16)^ (Figura 1):

#### Medidas de frequência: obtidas no eixo horizontal do fonetograma

- Frequência mínima: expressa em Hertz (Hz) e semitons (st), corresponde à nota mais grave produzida pelo participante.
- Frequência máxima: expressa em Hertz (Hz) e semitons (st), corresponde à nota mais aguda alcançada pelo participante.
- Extensão vocal: expressa em semitons (st), representa o número de semitons produzidos entre a frequência mínima e a frequência máxima.

#### Medidas de intensidade: obtidas no eixo vertical do fonetograma

- Intensidade mínima: expressa em decibéis (dB), corresponde à menor intensidade registrada entre as intensidades mínimas produzidas em toda a extensão vocal (curva inferior do fonetograma).
- Intensidade máxima: expressa em decibéis (dB), corresponde à maior intensidade registrada entre as intensidades máximas produzidas em toda a extensão vocal (curva superior do fonetograma).
- Extensão dinâmica máxima (EDM): expressa em decibéis (dB), representa a maior diferença entre as intensidades mínima e máxima em uma mesma frequência.

As curvas superior e inferior do fonetograma determinam o campo dinâmico ou perfil da extensão vocal dos participantes. Na Figura 1, observa-se que os registros verdes correspondem às emissões vocais registradas dos participantes. Quando o participante repete a mesma emissão, há sobreposição dos pontos, resultando em uma tonalidade verde escuro. O aspecto mais importante é o registro das intensidades nos limites máximos (curva superior) e mínimos (curva inferior) ao longo das frequências em toda a extensão vocal. Portanto, durante o exame da fonetografia, não é necessário que o indivíduo produza emissões na região central do campo dinâmico, pois todas as emissões abaixo da curva superior e acima da curva inferior são consideradas pertencentes ao seu campo dinâmico vocal.

### Análise dos dados

Os dados obtidos dos fonetogramas dos participantes dos grupos de fumantes (GF) e não fumantes (GNF) foram analisados por meio de porcentagens, médias e desvios-padrão.

A normalidade dos parâmetros extraídos do fonetograma foi feita pelo teste de *Shapiro-Wilk*.

A comparação entre os grupos GF e GNF, para cada parâmetro da fonetografia, foi realizada pelo Teste t de Student para amostras independentes adotando-se um nível de significância de 5%.

Além disso, foram realizadas as correlações entre o tempo de uso do tabaco e o número de cigarros consumidos por dia com os parâmetros obtidos pela fonetografia, por meio do Teste de correlação de Pearson, também com um nível de significância de 5%.

## RESULTADOS

### Caracterização da amostra

A caracterização dos participantes dos grupos GF e GNF encontra-se na Tabela 1. Ressalta-se que o tempo de tabagismo dos participantes do GF variou de 5 a 46 anos e o número de cigarros fumados por dia variou de 5 a 30 cigarros.

**Tabela 1:** Análise descritiva dos participantes do Grupo de Fumantes (GF) e do Grupo de Não Fumantes (GNF)

**Grupo Fumante**				**Grupo Não Fumante**	
**N**	**Idade (anos)**	**Tempo de tabaco (anos)**	**NºCigarros/dia**	**N**	**Idade (anos)**
1	20	5	5	1	18
2	21	5	20	2	23
3	21	6	7	3	21
4	23	6	20	4	21
5	24	7	15	5	26
6	21	7	20	6	21
7	23	8	10	7	22
8	25	8	10	8	23
9	26	8	10	9	25
10	27	8	20	10	26
11	28	10	10	11	28
12	26	10	20	12	24
13	27	11	10	13	26
14	33	14	5	14	34
15	29	15	20	15	30
16	32	15	20	16	32
17	31	17	20	17	32
18	31	18	10	18	30
19	47	18	20	19	45
20	48	20	5	20	49
21	40	22	5	21	39
22	37	22	15	22	37
23	50	24	5	23	50
24	41	24	10	24	42
25	57	24	15	25	58
26	54	34	15	26	54
27	49	35	15	27	49
28	50	35	30	28	52
29	54	38	12	29	54
30	58	43	20	30	57
31	58	46	20	31	59
**Média (DP)**	35 anos e 10 meses (21 anos 11 meses)	18 anos 6 meses (12 anos 10 meses)	14 (6)		36 anos 8 meses (23 anos 4 meses)

**Legenda:** n = número de participantes; DP = desvio-padrão

Os resultados evidenciaram uma forte correlação positiva entre a idade dos participantes do grupo de fumantes (GF) e o tempo de uso do tabaco (coeficiente = 0,929; p = 0,0001), indicando que a maior idade está diretamente associada ao maior tempo de tabagismo.

Por outro lado, não foi identificada correlação entre a idade dos participantes e o número de cigarros consumidos por dia (coeficiente = 0,156; p = 0,3934).

De modo semelhante, o tempo de uso do tabaco não apresentou correlação e o número de cigarros fumados por dia (coeficiente = 0,218; p = 0,3196).

### Fonetografia

Os parâmetros analisados pelo teste de normalidade de *Shapiro-Wilk* indicaram que os dados extraídos do fonetograma seguem a distribuição normal, conforme apresentado na Tabela 2.

**Tabela 2:** Distribuição das variáveis da Fonetrografia, do valor de p e da conclusão do Teste de Normalidade de *Shapiro-Wilk*

	Variáveis da Fonetografia	p	Conclusão do Teste
**Frequências**	Frequência Mínima (Hz)	0,132	Normal
	Frequência Máxima (Hz)	0,338	Normal
	Extensão Vocal	0,585	Normal
			
**Intensidades**	Intensidade Mínima (dB)	0,095	Normal
	Intensidade Máxima (dB)	0,085	Normal
	Extensão Dinâmica Máxima (dB)	0,250	Normal

**Legenda:** Hz = Hertz; dB = Decibel

Os participantes do Grupo de Fumantes (GF), quando comparados aos do grupo de não fumantes (GNF) apresentaram valores inferiores nas medidas de frequência (máxima e extensão vocal). Quanto às medidas de intensidade observou-se menor valor na extensão dinâmica máxima e maior valor na intensidade mínima (Tabela 3).

**Tabela 3:** Valores médios e desvios-padrão das medidas da intensidade e da frequência obtidos pela fonetografia dos participantes dos grupos de fumantes (GF) e de não fumantes (GNF) e a comparação entre os grupos

**MEDIDAS DA FONETOGRAFIA**		**GF Média (DP)**	**GNF Média (DP)**	**Valor p**
	**Mínima (Hz)**	84,3 (14,3)	86,4 (16,7)	0,696
**Frequências**	**Máxima (Hz)**	518,2 (166,3)	714,1 (262,8)	**0,004** ^*^
	**Extensão Vocal (st)**	31,5 (6,3)	36,9 (9,0)	0,005*
				
	**Mínima (dB)**	66,4 (7,3)	62,3 (5,7)	0,016*
**Intensidades**	**Máxima (dB)**	114,9 (6,4)	117,4 (6,6)	0,127
	**Extensão Dinâmica Máxima (dB)**	45,0 (9,4)	54,0 (9,2)	0,003*

* Valores significativos (p<0,05) - Teste t de Student para duas amostras independentes

**Legenda:** DP = Desvio-padrão; Hz = Hertz; St = Semitons; dB = Decibel

A Figura 2 ilustra a comparação entre o fonetograma de um participante do grupo de fumantes e o de um participante não fumante, ambos com idades semelhantes.

**Figura 2:** Tela dos fonetogramas de dois participantes do estudo: a) Fonetograma de um participante do Grupo de Fumantes (GF), com 58 anos de idade, fumante há 43 anos, com consumo diário de 20 cigarros. b) Fonetograma de um participante do Grupo de Não Fumantes (GNF) com 59 anos

Na análise de correlação entre o tempo de uso do tabaco e o número de cigarros por dia com os parâmetros da fonetografia dos participantes do grupo de fumantes (GF), observou-se correlação positiva entre o tempo de tabagismo e todas as medidas de frequência (mínima, máxima e extensão vocal), bem como com a intensidade mínima. Em relação ao número de cigarros consumidos por dia, foi identificada correlação positiva apenas com a frequência mínima (Tabela 4).

**Tabela 4:** Correlação do tempo de tabaco e do número de cigarros por dia com os parâmetros da frequência e da intensidade da fonetografia dos participantes do Grupo de Fumantes (GF)

	Frequências			Intensidades		
	Mínima	Máxima	Extensão vocal	Mínima	Máxima	Extensão Dinâmica Máxima
**Tempo Tabaco**	**-0,32 (0,024)** ^*^	-0,31 (0,006)*	-0,39 (0,043)*	0,03 (0,860)	-0,40 (0,033)*	-0,34 (0,908)
**Número Cigarros/dia**	-0,45 (0,016)*	0,07 (0,933)	0,21 (0,322)	0,13 (0,477)	0,01 (0,805)	-0,01 (0,943)

* Valores significativos (p<0,05) - Teste de correlação de *Pearson*

## DISCUSSÃO

O presente estudo buscou investigar os efeitos do uso do tabaco, do tempo de fumo e da quantidade de cigarros consumidos por dia no campo dinâmico da voz de homens fumantes, por meio da fonetografia. Os resultados obtidos confirmaram a hipótese inicialmente proposta. Contudo, não foram encontrados estudos anteriores que tenham utilizado essa ferramenta especificamente em indivíduos fumantes.

Na análise fonetográfica, a redução dos valores de frequência máxima e da extensão vocal observada nos participantes do Grupo de Fumantes (GF), em comparação aos do Grupo de Não Fumantes (GNF), pode ser atribuída ao impacto do tabagismo sobre o tecido das pregas vocais. Ayoub et al.^(10)^ e Pinto et al.^(22)^ relataram a diminuição da frequência fundamental na voz de indivíduos fumantes, decorrente do aumento da massa das pregas vocais. Essa alteração limita a amplitude de movimento das pregas vocais, resultando em menor capacidade de produzir sons em frequências mais agudas.

Em relação às medidas de intensidade obtidas na fonetografia, observou-se aumento significativo da intensidade mínima e redução da extensão dinâmica máxima no GF, em comparação ao GNF. Esse achado sugere que os fumantes apresentam dificuldade em manter a vibração das pregas vocais nos extremos da intensidade, o que pode ser atribuído à diminuição das forças aerodinâmicas da fonação, associada a alterações pulmonares e ao comprometimento respiratório causados pelo tabagismo^(12)^. De acordo com Boskabady et al.^(12)^, o cigarro impacta o controle vocal dos indivíduos fumantes.

A ampla variação do tempo de fumo entre os participantes do GF (5 a 46 anos) foi relevante para a análise da correlação entre o tempo de fumo e os parâmetros da fonetografia. A correlação negativa observada entre o tempo de fumo e todos os parâmetros relacionados à frequência indicou que, quanto maior o tempo de fumo, maior o deslocamento das frequências mínimas e máximas para um registro mais grave e menor a extensão vocal. Esses resultados corroboram achados da literatura^(10,14)^ que relataram redução da frequência fundamental e do pico cepstral em decorrência do espessamento e da inflamação da mucosa das pregas vocais.

Na correlação entre o tempo de fumo e as medidas de intensidade da fonetografia, observou-se correlação negativa entre o tempo de fumo e a intensidade máxima. Esse resultado corrobora estudos^(12,15)^ realizados com espirometria em fumantes, que indicaram que o hábito de fumar induz dependência química e, com o uso prolongado, compromete a capacidade respiratória, aumentando o esforço expiratório. Esses autores^(12,15)^ justificaram que a influência das toxinas do cigarro, acumuladas ao longo do tempo, reduzem a força do fluxo aéreo expiratório, necessária para manter a intensidade no seu limite máximo.

Na análise da correlação entre o número de cigarros consumidos por dia (5 a 30 cigarros/dia) e as medidas da fonetografia, observou-se correlação negativa entre o número de cigarros diários e a frequência mínima. Quanto maior o número de cigarros consumidos por dia, mais grave a frequência mínima da voz. Sabe-se que a redução da frequência vocal está relacionada ao aumento da massa das pregas vocais. Dessa forma, infere-se que o maior consumo diário de cigarros pode causar edema e espessamento da mucosa das pregas vocais. Um estudo que avaliou o aumento do volume de fumo evidenciou piores resultados na análise do pico cepstral na avaliação acústica vocal^(14)^.

A média registrada de 14 cigarros por dia entre os participantes do GF foi semelhante às médias relatadas em estudos com fumantes iranianos^(12)^ e americanos^(23)^ — 14 e 17 cigarros por dia, respectivamente —, o que indica um alto índice de consumo de cigarros nessas populações. No presente estudo, ressalta-se que nem a idade nem o tempo de tabagismo influenciaram a quantidade de cigarros consumidos. Chama a atenção o fato de que, embora o cigarro cause dependência^(15)^, o tempo de fumo não teve impacto sobre o número de cigarros fumados por dia.

Uma das limitações do presente estudo foi a ausência de verificação do histórico de exposição ao fumo passivo, uma vez que não foi considerada a possibilidade de os participantes do grupo controle frequentarem ambientes com fumantes — fator que poderia influenciar a qualidade vocal. Sugere-se que estudos futuros incluam e analisem essa variável.

Como recomendação para estudos futuros sugere-se verificar o campo dinâmico vocal em outros tipos de cigarros, como por exemplo de palha e eletrônico. Sugere-se ainda a especificação da raça dos participantes, uma vez que há poucos estudos que considerem a variação vocal de acordo com a raça.

A disseminação das informações sobre o impacto do cigarro no campo dinâmico da voz poderá desempenhar um papel crucial na ampliação do conhecimento de fonoaudiólogos e profissionais que trabalham com a saúde vocal. Além do fumo do cigarro convencional elevar o risco de câncer de laringe e de outras patologias e dos malefícios na voz, já consagrados pela literatura, a evidência da redução do campo dinâmico vocal em profissionais da voz — especialmente cantores — é de grande relevância, uma vez que esses profissionais utilizam a voz frequentemente nos limites máximos desse campo.

A fonetografia mostrou-se uma ferramenta útil como instrumento complementar nas avaliações da voz^(16-20)^, especialmente na população estudada. Seus resultados confirmaram as alterações vocais associadas ao tabagismo, corroborando os achados da literatura obtidos por meio de outros métodos de avaliação.

## CONCLUSÃO

O campo dinâmico vocal dos **homens fumantes** apresentou: 1) alterações significativas em relação aos homens **não fumantes** - valores menores da frequência máxima, da extensão vocal e da extensão dinâmica máxima e valores maiores de intensidade mínima; 2) quanto maior o tempo, menores os valores das frequências mínima e máxima, da extensão vocal e da intensidade máxima e 3) quanto maior a quantidade de cigarros consumidos/dia menor o valor da frequência mínima.

A fonetografia mostrou-se uma ferramenta útil como instrumento complementar nas avaliações da voz.
